# An AI-Driven Multimodal Sensor Fusion Framework for Fraud Perception in Short-Video and Live-Streaming Platforms

**DOI:** 10.3390/s26051525

**Published:** 2026-02-28

**Authors:** Ruixiang Zhao, Xuanhao Zhang, Jinfan Yang, Haofei Li, Zhengjia Lu, Wenrui Xu, Manzhou Li

**Affiliations:** 1 National School of Development, Peking University, Beijing 100871, China; 2 College of Economics and Management, Beijing University of Technology, Beijing 100124, China; 3 College of Information and Electrical Engineering, China Agricultural University, Beijing 100083, China

**Keywords:** multimodal perception modeling, multi-sensor fusion, cross-modal temporal attention, multimodal behavioral analytics, sequential signal processing

## Abstract

With the rapid proliferation of short-video platforms and live-streaming commerce ecosystems, marketing activities are increasingly manifested through complex multimodal sensing signals. These heterogeneous sensor data streams exhibit strong temporal dependency, high cross-modal coupling, and progressive evolutionary characteristics, making early-stage fraud perception particularly challenging for conventional unimodal or static analytical paradigms. Existing approaches often fail to effectively capture weak anomalous cues emerging across multimodal channels during the initial stages of fraudulent campaigns. To address these limitations, an artificial intelligence-driven multimodal sensor perception framework is proposed for temporal fraud detection in short-video environments. A multimodal temporal alignment module is first designed to synchronize heterogeneous sensor signals with inconsistent sampling granularities. Subsequently, a shared temporal encoding network is constructed to learn evolution-aware representations across multimodal sensor sequences. On this basis, a cross-modal temporal attention fusion mechanism is introduced to dynamically weight sensor contributions at different behavioral stages. Finally, a fraud evolution modeling and early risk prediction module is developed to characterize the progressive intensification of fraudulent activities and to enable risk assessment under incomplete temporal observations. Extensive experiments conducted on real-world datasets collected from multiple mainstream short-video platforms demonstrate the effectiveness of the proposed AI-driven sensing framework. The model achieves an overall accuracy of 0.941, precision of 0.865, recall of 0.812, and F1 score of 0.838, with the AUC further reaching 0.956, significantly outperforming text-based, vision-based, temporal, and conventional multimodal baselines. In early-stage detection scenarios utilizing only the first 30% of video content, the framework maintains stable performance advantages, achieving a precision of 0.812, recall of 0.704, and F1 score of 0.754, validating its capability for proactive fraud warning.

## 1. Introduction

With the rapid evolution of the digital economy and content platforms, live-streaming commerce, short-video marketing, and social media content distribution have gradually formed a highly integrated digital ecosystem. Within this ecosystem, multimodal perceptual data—including video frames, streamer speech, textual comments, user interaction behaviors, and transaction feedback—have become key observational sources for characterizing user decision-making processes and content dissemination mechanisms. Meanwhile, fraudulent marketing behaviors centered on “fake popularity,” “abnormal conversion,” and “coordinated manipulation” have increased simultaneously. Typical manifestations include inflated views and transactions, fake tipping, bot-generated comments, abnormal follower growth, and the manipulation of user financial decisions through emotional induction and scripted rhetoric. Such behaviors not only undermine the fairness of platform content distribution and recommendation mechanisms, but also weaken the trust foundation by misleading consumer cognition, thereby imposing higher requirements on platform governance and regulatory systems.

At both research and practical levels, detection methods for abnormal marketing and financial fraud have gradually shifted from static analysis relying on single-source textual data or transaction logs to comprehensive identification frameworks that integrate multi-source signals. However, in live-streaming commerce and short-video scenarios, fraudulent behaviors are often characterized by strong temporal dependency, intense interaction, and pronounced cross-modal coupling, which pose more systematic challenges to existing methods. First, significant discrepancies exist among different modalities—such as visual content, streamer speech, comment text, and user interaction behaviors—in terms of sampling frequency, temporal granularity, and semantic hierarchy, making it difficult to align multimodal information along the temporal dimension and form unified perceptual evidence. Second, abnormal marketing behaviors usually emerge in the form of weak signals and progressive evolution, rendering early-stage identification difficult through single-modality or instantaneous observations. Third, differences across platform formats, streamer types, and product categories further introduce distribution shifts, leading to insufficient generalization capability when models are deployed across scenarios. From the perspective of methodological development in perception and modeling, deep representation learning has established relatively mature technical paradigms in single-modality perceptual tasks such as text, vision, and audio. For example, transformer-based sequence modeling and attention mechanisms have demonstrated significant advantages in capturing long-range dependencies and global interaction patterns, providing fundamental components for unified modeling of multimodal temporal signals [1]. In the textual modality, pre-trained language models represented by BERT and RoBERTa acquire stable semantic representations through self-supervised learning, enabling more reliable perception of emotional tendencies, persuasive expressions, and strategic rhetoric in comment text [2,3]. In the visual modality, ResNet and ViT represent convolution-based and self-attention-based backbone architectures, respectively, and are capable of extracting discriminative visual cues from product presentation styles, streamer actions, and scene variations. Among them, ViT and its improved variant Swin further enhance perceptual capability for complex scenes through global modeling or hierarchical window-based attention mechanisms [4,5,6]. In addition, vision–language alignment frameworks such as CLIP and ALIGN map images and text into a shared embedding space via contrastive learning, providing a general paradigm for cross-modal consistency modeling [7,8].

However, unlike traditional content understanding tasks, abnormal marketing and financial fraud in live-streaming commerce are not solely reflected by inconsistencies at the content level, but are more prominently manifested as abnormal coordination and evolutionary patterns of multimodal signals along the temporal dimension. Fraudulent behaviors often undergo a process from weak cues and strategic priming to concentrated outbreaks, accompanied by group-level coordination and interaction amplification. This imposes higher requirements on models in terms of cross-modal alignment, temporal perception, and structured modeling.

Motivated by these observations, the problem of financial fraud detection in live-streaming commerce and short-video scenarios is re-examined from the perspective of multimodal temporal perception. Short-video content is regarded as a multimodal sensor-based temporal data stream, in which visual, audio, and textual signals are jointly modeled along a unified timeline to characterize the dynamic evolution of financial fraud behaviors. On this basis, a multimodal temporal perception-driven deep learning framework is constructed to enable accurate identification and early warning of financial fraud behaviors on social media platforms.

The main contributions of this paper can be summarized as follows:

1. From the perspective of multimodal temporal perception, the problem of financial fraud detection on short-video platforms is systematically formulated by abstracting short-video content as a multimodal sensor-based temporal data stream.
2. A mathematically novel monotonic cross-modal attention mechanism is proposed to jointly model visual, audio, and textual multimodal information along a unified timeline. By introducing a mathematically rigorous monotonic constraint, this mechanism ensures strict non-decreasing temporal alignment across asynchronous modalities, effectively capturing the temporal dynamics of fraudulent behaviors.
3. We establish theoretical guarantees for the proposed monotonic constraint, demonstrating that it provably improves the stability of cross-modal phase shifts and bounds the gradient variance during long-sequence training, thereby addressing the instability inherent in standard unconstrained temporal attention.
4. Extensive experiments demonstrate the effectiveness of the proposed method in terms of financial fraud detection performance, early identification capability, and model robustness.

## 2. Related Work

### 2.1. Financial Fraud Detection in Social Media and Short-Video Platforms

With the enrichment of platform data modalities, user behavior and relational structure information have gradually been incorporated, and fraud identification problems have been modeled as anomaly detection or risk prediction tasks. Under high-noise and non-stationary distribution conditions, traditional methods based on statistical assumptions, distance measures, or density estimation have shown limited stability, which has promoted deep learning approaches as the mainstream choice [9]. Although representation learning has been shown to be effective in capturing complex anomalous patterns, challenges remain in multimodal, high-dimensional, and strongly temporal-dependent scenarios. In the domain of marketing fraud and platform governance, graph-structured information has been widely utilized to characterize interactions and coordinated behaviors among users. It has been pointed out that graph anomaly detection requires simultaneous consideration of attribute anomalies and structural anomalies, and that deep representation learning holds potential for capturing complex relational patterns [10]. In recent years, graph neural networks have been extensively applied to financial fraud detection tasks due to their ability to aggregate neighborhood information through message passing mechanisms [11]. CARE-GNN introduces similarity-driven neighbor selection to mitigate neighborhood contamination caused by feature and relation camouflage [12]. PC-GNN addresses extreme class imbalance through improved sampling and training strategies [13]. SCN_GNN further strengthens structural and attribute similarity modeling to improve discrimination stability under weak supervision [14]. In addition, research in comment and content manipulation scenarios has gradually shifted from simple to intention recognition and contextual perception. For example, Hou et al. construct a multimodal framework integrating text, images, and user profiles to identify fake review intentions, demonstrating the effectiveness of multimodal context for abnormal marketing behavior detection [15]. Overall, although notable progress has been achieved in text-, behavior-, and structure-based financial fraud detection, existing methods still struggle to adequately characterize the temporal evolution of multimodal fraudulent behaviors in short-video scenarios.

### 2.2. Multimodal Content Analysis and Cross-Modal Learning Methods

With the success of transformer architectures in sequence modeling, multimodal learning has gradually shifted toward unified modeling frameworks based on attention mechanisms. Self-attention enables the modeling of associations among textual tokens, visual patches, and cross-modal elements across temporal and semantic dimensions, allowing questions such as whether visual content supports persuasive rhetoric or whether comments are consistent with displayed content to be modeled through attention distributions and similarity structures [1]. Meanwhile, the pre-training–fine-tuning paradigm significantly reduces the dependence of multimodal models on downstream labeled data, making them more suitable for risk recognition tasks with scarce labels and rapidly evolving strategies. In the textual modality, BERT learns bidirectional contextual representations through masked language modeling, enabling stable capture of complex semantics such as metaphor, exaggeration, and persuasive expressions [2]. RoBERTa further enhances robustness by optimizing training strategies and enlarging training corpora [3]. In the visual modality, ResNet alleviates deep network degradation through residual connections and has become a general-purpose backbone for vision tasks [4]. ViT encodes images as patch sequences using transformers, enabling global relation modeling via unified attention mechanisms and demonstrating strong representation capacity for complex live-streaming scenes [5]. Swin introduces hierarchical window-based attention to achieve efficient multi-scale modeling with a favorable balance between performance and computational cost [6]. For cross-modal alignment, CLIP and ALIGN perform contrastive learning on large-scale image–text pairs to embed visual and textual information into a shared space, enabling direct measurement of cross-modal semantic consistency and demonstrating strong zero-shot transfer capability [7,8]. In fusion structure design, models such as MMBT, ViLBERT, LXMERT, and UNITER explicitly model visual–language interactions through unified or interactive representation spaces [16,17,18,19]. Although these studies provide valuable paradigms for multimodal risk recognition, most of them focus on static content understanding and pay limited attention to temporal dynamics across modalities.

### 2.3. Temporal Modeling and Behavioral Evolution Perception Methods

In short-video and live-streaming commerce scenarios, financial fraud and abnormal marketing behaviors rarely occur instantaneously, but instead exhibit gradual temporal evolution. Therefore, effective temporal modeling of multimodal behavioral sequences has become a key issue for understanding and identifying fraudulent activities. Modeling approaches based on temporal neural networks and attention mechanisms provide essential tools for capturing behavioral evolution [20,21]. Anomaly detection studies indicate that under high-noise and non-stationary distributions, point-wise discrimination methods are often unstable, whereas temporal modeling can capture trajectories of behavioral transitions from normal to abnormal states [9]. The introduction of attention mechanisms into temporal modeling enables dynamic focusing on critical time segments, thereby enhancing sensitivity to weak signals and delayed anomalies. Meanwhile, in relational data, anomalous behaviors are frequently accompanied by group-level coordination and structural changes, making temporal graph modeling an important direction for understanding complex manipulation behaviors [10]. In recent years, graph neural networks have been extensively applied to financial fraud detection due to their ability to aggregate neighborhood information and model individuals within evolving relational structures. Structural information not only complements individual features but also reflects the accumulation and amplification of coordinated manipulation, relation camouflage, and class imbalance over time. A survey by Motie and Raahemi highlights that although GNNs effectively capture complex relational patterns in transaction networks, challenges remain in label scarcity, class imbalance, heterogeneous relation modeling, and insufficient temporal dynamics [11]. To address these issues, CARE-GNN mitigates neighborhood contamination through similarity-driven neighbor selection [12], PC-GNN improves minority class learnability under extreme imbalance via a “pick and choose” sampling strategy [13], and SCN_GNN enhances structural and attribute similarity modeling to reduce noise interference during dynamic aggregation [14]. In comment and content manipulation scenarios, related studies have also shifted from static toward perception of intention evolution with contextual accumulation. For instance, Hou et al. propose a multimodal framework integrating text, images, and user profiles to identify fake review intentions within comment contexts, and experimental results demonstrate that incorporating multimodal context significantly improves discrimination of deceptive intentions [15]. These findings suggest that abnormal marketing behavior recognition should not rely solely on instantaneous content features, but should instead integrate progressively accumulated contextual information across content publication and user interaction processes to understand the formation and reinforcement of manipulation intent over time.

## 3. Materials and Method

### 3.1. Data Collection

The short-video financial fraud dataset constructed in this study is collected from several mainstream public short-video and content community platforms, including Douyin (operated by ByteDance, Beijing, China), Kuaishou (Kuaishou Technology, Beijing, China), and Xiaohongshu (Xingin Information Technology Co., Ltd., Shanghai, China). The data collection period spans from June 2023 to December 2023, covering seven consecutive months of content publishing and user interaction activities, so as to ensure temporal continuity and integrity of behavioral patterns. Data collection is strictly limited to publicly accessible information provided by the platforms and is conducted through compliant application programming interfaces and automated crawling tools, without involving any user privacy data or non-public information. The collected samples focus on finance-related short-video content, mainly including videos associated with investment promotion, profit promises, course marketing, and related persuasive marketing rhetoric, as shown in Table 1.

For the visual modality, complete short-video files are collected, resulting in a total of 18,742 video samples, with video durations primarily ranging from 15 s to 180 s. To preserve temporal information integrity, frame-level sampling is performed at fixed intervals, with one frame extracted per second and full timestamp information retained. This design enables effective characterization of temporal evolution in visual patterns such as streamer appearance, product presentation, subtitle changes, and scene transitions. The audio modality is synchronously extracted from the corresponding videos, retaining the original sampling rate of 16 kHz and continuous speech streams, which are used to model temporal characteristics such as speech rhythm, intonation variation, and emotional fluctuation. The textual modality consists mainly of video titles, content descriptions, and user comments. In total, 2,316,584 comment texts are collected and organized according to publishing time and comment timestamps, so as to reflect the temporal dynamics of user feedback intensity and topic evolution. For sample annotation, fraud and non-fraud labels are assigned at the video level by jointly considering publicly available platform penalty announcements, content moderation indicators, and manual verification results. The final dataset contains 3964 fraud samples and 14,778 non-fraud samples. Annotation is performed at the video level, and consistency across visual, audio, and textual data corresponding to the same video is strictly maintained along the temporal dimension. By aligning multimodal observations along a unified timeline, the constructed dataset exhibits high consistency in terms of multimodal completeness and temporal continuity, and can therefore serve as a standard input for multimodal temporal perception-based financial fraud detection tasks.

### 3.2. Data Preprocessing and Augmentation

To facilitate effective multimodal temporal modeling for short-video financial fraud detection, we perform standardized preprocessing, temporal segmentation, and cross-modal alignment for the visual, audio, and textual signals.

For the visual modality, raw short videos are decoded into frame sequences and uniformly sampled. Given a video duration of *T* seconds and a sampling rate of *r* frames per second, we obtain a frame sequence $V={\left\{v_{t}\right\}}_{t=1}^{L}$, where L=⌊Tr⌋. To balance early-stage detection with full-sequence analysis, we apply sliding-window temporal segmentation with a window length of *w* frames and a stride of *s* frames. This yields a segment set $S={\left\{V_{i}\right\}}_{i=1}^{N_{s}}$, where $V_{i}=\left\{v_{(i-1)s+1},\cdots ,v_{(i-1)s+w}\right\}$ and $N_{s}=\left\lfloor \frac{L-w}{s}\right\rfloor +1$. Each frame undergoes scale normalization and center cropping. Furthermore, video-level normalization is applied to mitigate domain shifts caused by platform-specific compression and resolution differences. To maintain temporal continuity, missing or corrupted frames are handled via temporal neighborhood interpolation or segment dropping.

For the audio modality, raw speech streams are extracted synchronously and resampled to 16 kHz. This is followed by pre-emphasis, voice activity detection, and energy normalization to suppress background noise. To extract stable time-frequency representations, we employ the short-time Fourier transform. Given an audio signal x[n] and a window function h[·], the representation is computed as $X\left(\tau ,\omega \right)=\sum_{n}x\left[n\right]h\left[n-\tau \right]e^{-j\omega n}$. This is subsequently mapped to a log-Mel spectrogram $A\in R^{T_{a}\times F}$, where $T_{a}$ is the number of time frames and *F* denotes the Mel frequency bands. To temporally align the audio with the visual segments, the spectrogram is segmented using an identical windowing mechanism, and linear interpolation is applied to synchronize audio time steps with visual time steps.

For the textual modality, metadata and user comments undergo deduplication, sentence segmentation, and noise filtering to remove explicit advertising templates, meaningless symbols, and hyperlink-only segments. Comments are sorted chronologically to form a sequence $U={\left\{u_{t}\right\}}_{t=1}^{T_{u}}$. Each text instance is tokenized into subwords and truncated to a maximum length. We utilize a pre-trained language model to extract contextual semantic representations. Let f(·) denote the text encoder; the representation for the *t*-th text is $e_{t}=f\left(u_{t}\right)\in R^{d}$. These features are then mapped onto the unified timeline. To mitigate cross-platform linguistic variations and colloquial noise, we introduce a confidence-based filtering threshold for low-quality texts and apply random masking during training to enhance robustness against missing comments.

Finally, we employ specific data augmentation and re-weighting strategies to address class imbalance and improve model generalization. To counter the scarcity of fraud samples, cost-sensitive learning is implemented via a weighted cross-entropy loss defined as $L=-\sum_{c\in \{0,1\}}w_{c}\;y_{c}\log p_{c}$, where $w_{1}>w_{0}$ amplifies the gradient contribution of the minority fraud class. Furthermore, to enhance robustness against real-world noise and platform variations, we introduce modality-specific perturbations. These include temporal jittering, segment cropping, and mild frame dropping in the visual modality to simulate platform lag and editing variations; time stretching and additive noise in the audio modality to simulate diverse recording environments; and synonym replacement and random deletion in the textual modality to reflect natural colloquial fluctuations. Crucially, all multimodal augmentations strictly adhere to a temporal consistency principle, where perturbations are applied synchronously across all modalities within the same temporal window. This preserves cross-modal alignment, effectively expanding the training distribution while maintaining the semantic integrity of the fraud signals.

### 3.3. Proposed Method

#### 3.3.1. Overall Architecture

Following standardized preprocessing, each short-video sample is formulated as a tri-modal temporal input aligned along a unified timeline. This input comprises a visual frame sequence, a synchronized audio feature sequence, and a timestamp-ordered textual sequence, which are jointly fed into an end-to-end risk perception model. Initially, the three modalities are independently processed by their respective low-level feature encoders. These encoders map the raw observations into modality-specific temporal representations with consistent dimensionality. Subsequently, a multimodal temporal alignment module resamples and projects signals of varying sampling rates onto unified time steps. This ensures that visual, audio, and textual evidence is simultaneously accessible at each step, thereby establishing temporal comparability and aggregatability across modalities. Building upon this, a shared temporal encoding module performs sequence modeling over the aligned multimodal sequences using shared parameters. This module captures modality-internal dynamic patterns through a consistent temporal perception mechanism, generating modality-level latent representations at each time step. Next, a cross-modal temporal attention fusion module dynamically weights these latent representations. The learned attention weights adaptively capture the varying contributions of different modalities to fraud identification at different temporal stages, ultimately generating a unified fused temporal representation. This design ensures that critical evidence related to persuasive rhetoric, visual cues, and interaction semantics is selectively emphasized as the fraudulent behavior evolves. Finally, a fraud evolution modeling and early risk prediction module performs sequential modeling and stage-wise aggregation on the fused representations. This module produces a risk state representation that reflects the overall behavioral trajectory and outputs stage-level risk scores at arbitrary temporal truncation points. Consequently, the framework supports both comprehensive video-level fraud discrimination and early-stage risk warning during content dissemination. During training, video-level labels serve as supervisory signals to jointly optimize all modules. This forms a closed-loop coupling among temporal alignment, cross-modal fusion, and evolution modeling, thereby enabling the accurate identification and early prediction of short-video financial fraud. The entire process is shown in Algorithm 1.
**Algorithm 1** Multimodal Temporal Framework for Financial Fraud Detection**Input:** Raw visual sequence *V*, raw audio signal *A*, raw textual sequence *U*, target sequence length *L*, scaling temperature $\tau =\sqrt{d_{k}}$.
**Output:** Fraud probability score *P*.
 1: **// Step 1: Data Preprocessing & Modality-Specific Encoding**
 2: $F_{v}\leftarrow \mathrm{VisualEncoder}\left(\mathrm{Process}\left(V\right)\right)$ {Extract frame-level visual features}
 3: $F_{a}\leftarrow \mathrm{AudioEncoder}\left(\mathrm{LogMel}\left(\mathrm{STFT}\left(A\right)\right)\right)$ {Extract audio time-frequency features}
 4: $F_{t}\leftarrow \mathrm{TextEncoder}\left(\mathrm{Tokenize}\left(U\right)\right)$ {Extract contextual textual features}
 5:  
 6: **// Step 2: Multimodal Temporal Alignment**
 7: **for** m∈{v,a,t} 
**do**
 8:     ${\tilde{F}}_{m}\leftarrow \mathrm{TemporalAlign}\left(F_{m},L\right)$ {Interpolate/Resample to unified timeline *L*}
 9: **end for**
10:
11: **// Step 3: Shared Temporal Encoding**
12: **for** m∈{v,a,t} 
**do**
13:     $H_{m}\leftarrow \mathrm{SharedTemporalEncoder}\left({\tilde{F}}_{m}\right)$ {Capture modality-internal dynamics}
14: **end for**
15:
16: **// Step 4: Cross-Modal Temporal Attention Fusion**
17: $H_{fused}\leftarrow \emptyset$
18: **for** 
t=1 
*L* 
**do**
19:     Apply monotonic constraint mask *M* to prevent future-information leakage
20:     $W_{attn}\leftarrow \mathrm{Softmax}\left(\frac{Q_{t}K_{t}^{⊤}}{\tau }⊙M\right)$ {Calculate temperature-scaled attention weights}
21:     $H_{fused}^{\left(t\right)}\leftarrow W_{attn}\cdot V_{t}$ Dynamically fuse representations across modalities
22: **end for**
23:
24: **// Step 5: Fraud Evolution Modeling & Early Risk Prediction**
25: $S\leftarrow \mathrm{EvolutionModeling}(H_{fused})$ {Perform stage-wise temporal aggregation}
26: P←RiskPredictor(S) {Generate overall and early-stage risk scores}
27: **return** *P*


#### 3.3.2. Multimodal Temporal Alignment and Shared Temporal Encoding Module

**Figure 1:**
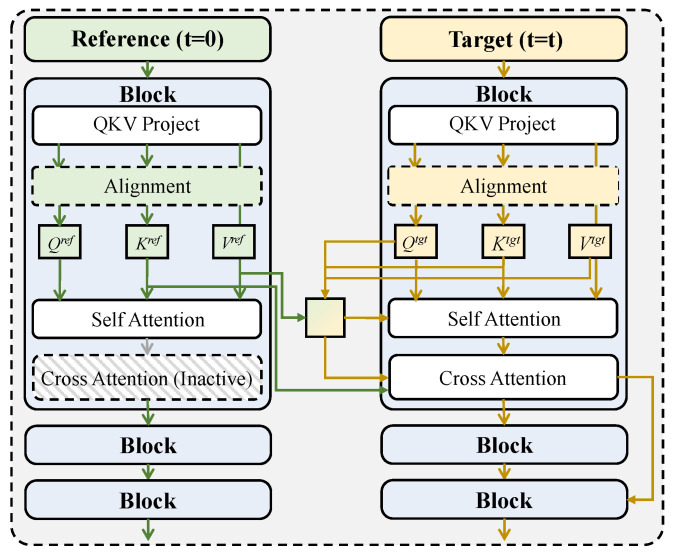
Illustration of the multimodal temporal alignment and shared temporal encoding module.

In the multimodal temporal alignment and shared temporal encoding module, the visual, audio, and textual sequences that have completed low-level feature extraction are treated as three heterogeneous temporal signals. The core objective is to achieve strict alignment and comparable modeling across modalities on a unified timeline while preserving the intrinsic temporal structure of each modality, as shown in Figure 1.

The encoded multimodal inputs are denoted as $X^{v}\in R^{T_{v}\times C_{v}}$, $X^{a}\in R^{T_{a}\times C_{a}}$, and $X^{t}\in R^{T_{t}\times C_{t}}$, where $T_{\cdot }$ represents the number of temporal steps and $C_{\cdot }$ denotes the feature dimension of each modality. Since $T_{v}\neq T_{a}\neq T_{t}$ due to different sampling mechanisms, direct cross-modal fusion would result in temporal misalignment and information distortion. To address this issue, a learnable temporal mapping function is introduced to remap the three modality sequences onto a shared timeline of length *T*. Specifically, the timeline length *T* is not fixed as a static constant, but is dynamically determined based on the base sampling rate *r* and the video duration Tr. To efficiently process these variable-length sequences within unified batches, zero-padding and attention masking mechanisms are applied to handle unequal sequence lengths. This process is jointly implemented through linear temporal projection and interpolation operators, which can be expressed as

(1) $${\tilde{X}}_{\tau }^{m}=\sum_{i=1}^{T_{m}}\omega_{\tau ,i}^{m}\;X_{i}^{m},\;\sum_{i}\omega_{\tau ,i}^{m}=1,\;m\in \left\{v,a,t\right\},$$

where the weights $\omega_{\tau ,i}^{m}$ are determined by relative temporal distance functions together with learnable temperature parameters. This design guarantees continuity and differentiability of the temporal resampling process, allowing automatic adaptation to different temporal resolutions of each modality during training. After temporal alignment, the three modalities are uniformly projected to the same channel dimension C=256 and fed into a shared temporal encoding network. This network adopts a stacked transformer encoder architecture with a total depth of L=4, where each layer consists of multi-head self-attention and feed-forward transformations. The hidden width is fixed at $d_{\mathrm{model}}=256$, the number of attention heads is set to h=8, and the dimension of each head is 32. For any time step τ, identical parameters are applied to all modalities in the shared encoder, and the state update is formulated as

(2) $$H_{\tau ,l}^{m}=\mathrm{FFN}\;\left(\mathrm{MHSA}\;\left(H_{\tau ,l-1}^{m}\right)\right),\;l=1,\ldots ,L,$$

where $H_{\tau ,0}^{m}={\tilde{X}}_{\tau }^{m}$. Parameter sharing enforces consistent temporal modeling operators across modalities, which is mathematically equivalent to constraining multimodal mappings within the same function space, thereby reducing representation bias introduced by temporal scale differences. It can be shown that, under shared parameters, if modality sequences correspond to the same latent event at a given time step, their hidden representations converge in expectation to the same temporal subspace, i.e., there exists a mapping Φ such that $E\left[H_{\tau ,L}^{v}\right]\approx E\left[H_{\tau ,L}^{a}\right]\approx E\left[H_{\tau ,L}^{t}\right]=\Phi \left(\tau \right)$, which provides a theoretical foundation for subsequent cross-modal fusion. Furthermore, to enhance temporal positional expressiveness, rotary relative positional embeddings are incorporated into the shared encoding process, explicitly injecting temporal indices into attention computation so that semantic differences of identical content across different temporal stages can be distinguished. The temporally modulated attention is computed as

(3) $$\mathrm{Attn}\left(Q,K,V\right)=\mathrm{softmax}\;\left(\frac{\left(QR_{\tau }\right){\left(KR_{\tau }\right)}^{⊤}}{\sqrt{d_{k}}}\right)V,$$

where $R_{\tau }$ denotes the rotary positional matrix associated with time step τ. This mechanism preserves sensitivity to temporal order without relying on absolute sequence length, which facilitates generalization across videos of varying durations. The multimodal temporal alignment and shared temporal encoding module is tightly coupled with the subsequent cross-modal temporal attention module. The former produces modality-level temporal representations that are structurally and scale-consistent along a unified timeline, while the latter performs dynamic modality weighting based on these representations. This design avoids introducing additional temporal compensation terms during attention fusion, thereby reducing model complexity. For financial fraud detection, the proposed module enables early-stage alignment of weak risk signals across visual, audio, and textual modalities, synchronously amplifying multimodal responses to the same fraudulent event and providing a stable and interpretable temporal foundation for subsequent evolution modeling and early risk prediction.

#### 3.3.3. Cross-Modal Temporal Attention Fusion Module

**Figure 2:**
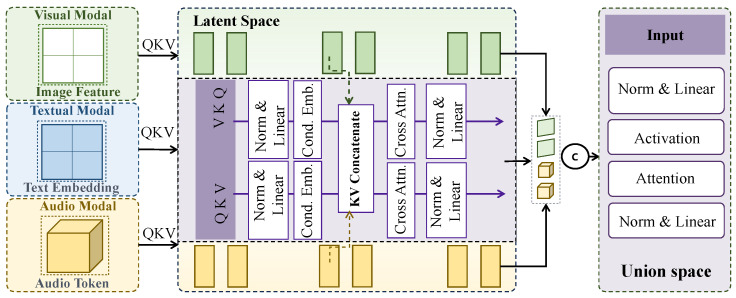
Illustration of the cross-modal temporal attention fusion module.

In the cross-modal temporal attention fusion module, the design objective is not to model temporal dependencies within a single sequence, but to explicitly characterize the relative contribution differences of different modalities to fraud identification at the same temporal stage. Therefore, this module is functionally distinct from standard self-attention mechanisms. Self-attention focuses on modeling correlations among different time steps or tokens within the same sequence, where attention weights are derived from a homogeneous representation space. In contrast, cross-modal temporal attention operates under the premise of temporal synchronization and modality heterogeneity, and performs competitive weighting over modality-specific representations at a fixed time step. As shown in Figure 2, after temporal alignment and shared temporal encoding, latent representations from visual, audio, and textual modalities at time step τ are obtained as $h_{\tau }^{v}$, $h_{\tau }^{a}$, and $h_{\tau }^{t}\in R^{C}$, where the channel dimension is uniformly set to C=256. Attention computation is conducted along the modality dimension rather than the temporal dimension, such that the model focuses on determining which modality is more reliable and discriminative at the current stage, fundamentally differing from self-attention mechanisms operating over time or space.

Structurally, the cross-modal temporal attention fusion module consists of two stacked cross-modal attention units, each composed of modality-level attention mapping and linear reconstruction. For a given time step τ, modality representations are first concatenated into a modality set $H_{\tau }=\left[h_{\tau }^{v};h_{\tau }^{a};h_{\tau }^{t}\right]\in R^{3\times C}$, which is then linearly projected to generate queries, keys, and values with a projection width of *C*. Specifically, the shared query vector $q_{\tau }$ is generated by applying a learnable linear projection to the mean-pooled representation of the three modalities at time step τ, acting as a comprehensive multimodal context for the current stage. Meanwhile, the key and value vectors are obtained through modality-specific linear projections of each individual modality representation. Cross-modal attention weights are computed as

(4) $$\alpha_{\tau }^{m}=\frac{\exp\left(q_{\tau }^{⊤}k_{\tau }^{m}/\sqrt{C}\right)}{\sum_{m^{\prime }\in \left\{v,a,t\right\}}\exp\left(q_{\tau }^{⊤}k_{\tau }^{m^{\prime }}/\sqrt{C}\right)},$$

where $q_{\tau }$ denotes the shared query vector and $k_{\tau }^{m}$ denotes the key vector of modality *m*. The fused temporal representation is obtained through modality-weighted summation,

(5) $$z_{\tau }=\sum_{m\in \{v,a,t\}}\alpha_{\tau }^{m}v_{\tau }^{m},$$

and is then passed to the next attention unit through residual connections and layer normalization. The two-layer stacked design enables the model to capture both direct modality competition and higher-order nonlinear patterns of modality importance variation across temporal stages, without introducing excessive depth that could destabilize training.

The cross-modal temporal attention module complements the preceding shared temporal encoding module. While shared encoding ensures temporal-semantic alignment and scale consistency across modalities, cross-modal attention performs modality-level information selection on top of this alignment. Under the temporal alignment assumption, if a modality carries higher discriminative information at time step τ, the expected inner product between its key vector and the query vector is larger, leading to a higher normalized attention weight after softmax. Thus, this mechanism can be interpreted as an adaptive maximum-likelihood weighting of multimodal evidence at each time step. This design is particularly critical for financial fraud scenarios, where fraudulent behaviors often evolve from rhetorical persuasion to interaction amplification, and the discriminative power of different modalities varies across stages. Through cross-modal temporal attention fusion, textual and audio cues are automatically emphasized in early stages, while interaction and visual anomalies are amplified in later stages, thereby providing more focused and interpretable temporal representations for subsequent fraud evolution modeling and early risk prediction.

#### 3.3.4. Fraud Evolution Modeling and Early Risk Prediction Module

**Figure 3:**
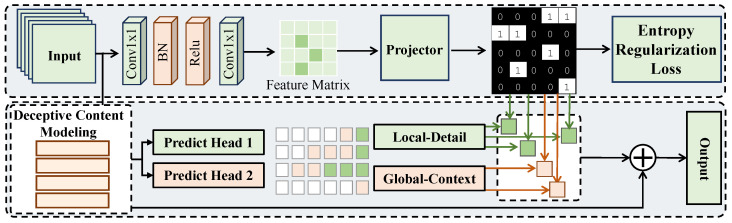
Illustration of the fraud evolution modeling and early risk prediction module.

In the fraud evolution modeling and early risk prediction module, the objective is to characterize the evolution of multimodal fraud signals from weak to strong and from local to global over time, and to provide stable risk judgments at early stages when information is not yet fully exposed. As shown in Figure 3, this module takes the cross-modally fused temporal representations from the previous stage as input, denoted as $Z=\{z_{1},\ldots ,z_{T}\}$, where each time step has a feature dimension of C=256. The overall structure follows a design paradigm of gated selection, multi-expert temporal modeling, and stage-wise risk output, enabling joint modeling of local detail sensitivity and global evolution perception.

First, a lightweight gating network is introduced to evaluate the fused features at each time step and determine the participation degree of different modeling experts. The gating network consists of two one-dimensional convolutional layers with kernel size 1, mapping channels from 256 to 64 and then to 2, with batch normalization and ReLU activation applied in between, yielding a soft gating probability vector $g_{\tau }\in R^{2}$ at time step τ. This vector reflects whether the current stage is more inclined toward local anomaly amplification or global trend accumulation. During training, a straight-through estimator with a temperature parameter is used to map the soft gate into an approximately discrete selection mask $m_{\tau }$, formulated as

(6) $$m_{\tau }=\mathrm{STE}\left(g_{\tau }>\theta \right),$$

where θ is an adjustable threshold. The straight-through estimator preserves differentiability of discrete decisions during backpropagation. To prevent gate collapse, an entropy-based regularization term is introduced on the gating distribution, ensuring sufficient activation of different experts along the temporal dimension. In the expert modeling stage, two parallel temporal expert networks with independent structures but shared inputs are constructed. The local-detail expert adopts a two-layer one-dimensional temporal convolution with kernel sizes 3 and 5, and channel dimensions expanding from 256 to 512 and then reducing back to 256, thereby enhancing sensitivity to short-term abnormal fluctuations. The global-context expert employs a three-layer stacked autoregressive temporal encoding structure with a hidden width of 256, which is designed to model long-range evolutionary trends. For any time step τ, the two experts output state representations $s_{\tau }^{\mathrm{loc}}$ and $s_{\tau }^{\mathrm{glob}}$, which are combined through gated weighting as

(7) $$s_{\tau }=m_{\tau ,1}s_{\tau }^{\mathrm{loc}}+m_{\tau ,2}s_{\tau }^{\mathrm{glob}}.$$

It can be shown that when abnormal signals exhibit abrupt changes within a short interval, the local expert receives a higher expected gradient contribution, whereas when fraud behaviors evolve through gradual accumulation, the global expert dominates the final representation, enabling adaptive modeling of diverse fraud patterns. In the risk prediction stage, each temporal evolution state $s_{\tau }$ is mapped to a stage-wise risk score ${\hat{y}}_{\tau }$ through a fully connected layer with input dimension 256 and output dimension 1, followed by a sigmoid function constraining outputs to [0,1]. Since fraud evolution exhibits a statistically monotonic intensification property, a temporal consistency constraint is imposed during training, encouraging the predicted risk sequence to satisfy ${\hat{y}}_{\tau +1}\geq {\hat{y}}_{\tau }$ in expectation. This constraint is implemented by penalizing violations of monotonicity, guiding the model to learn evolution-consistent risk trajectories. This module operates in close synergy with the preceding cross-modal temporal attention fusion module. While cross-modal attention selects the most discriminative modality evidence at each time step, fraud evolution modeling further explains how such evidence accumulates over time and is transformed into risk judgments. From a theoretical perspective, the gated multi-expert structure is equivalent to constructing a piecewise approximation of the risk function over the temporal sequence, enabling stable estimation under limited observations. In practical applications, this design substantially enhances sensitivity to weak fraud signals at early stages while avoiding delayed identification caused by over-reliance on isolated strong cues, making it particularly suitable for early warning of short-video financial fraud scenarios.

## 4. Results and Discussion

### 4.1. Experimental Settings

Given that the dataset was collected over a consecutive seven-month period across multiple mainstream platforms, we adopt a strict temporal data splitting strategy to evaluate model robustness and practical temporal generalization. Specifically, the dataset is partitioned chronologically: data from the first five months are allocated for training, data from the sixth month is used as the validation set, and data from the seventh month is reserved for testing.

All experiments are conducted under a unified deep learning computing environment, and both model training and evaluation are implemented based on the PyTorch framework (PyTorch v2.1.0, Meta AI, Menlo Park, CA, USA). The hardware platform consists of a high-performance server equipped with an NVIDIA RTX 4090 GPU with 24GB of video memory, an Intel Xeon Gold series CPU, and 256GB of system memory, which supports efficient parallel processing of multimodal temporal data. The software environment is based on the Ubuntu 20.04 operating system, with CUDA 11.8 and cuDNN 8.x.

During model training, the AdamW optimizer is adopted, with the initial learning rate set to $1\times 10^{-4}$ and the weight decay coefficient set to $1\times 10^{-5}$. The batch size is fixed at 16, and the maximum number of training epochs is set to 50, while an early stopping strategy is applied on the validation set to prevent overfitting. The dimensionality of multimodal features is uniformly set to 256, the number of Transformer encoding layers is set to 4, and the number of attention heads is set to 8. All remaining hyperparameters are tuned on the validation set to ensure experimental fairness and result stability. Furthermore, it is important to clarify that to maintain rigorous evaluation consistency, the exact same fully trained model is utilized for both full-sequence evaluation and early-stage truncated inference, without any separate retraining or parameter fine-tuning.

### 4.2. Baselines and Evaluation Metrics

To systematically evaluate the performance of the proposed multimodal temporal fraud perception model, several representative baseline methods are selected for comparison, covering different technical paradigms including unimodal modeling, multimodal fusion, and temporal modeling. For textual baselines, a pretrained language model–based BERT [22] is adopted, which is capable of stably capturing semantic and emotional cues in comments and promotional scripts through deep contextual modeling. For visual baselines, ResNet [23] and ViT [24] are employed, representing mainstream paradigms of convolutional networks and self-attention architectures for video content modeling, respectively, and enabling effective extraction of discriminative visual information such as scene structure, presenter behavior, and product presentation. Multimodal baselines include an early fusion method based on feature concatenation [25] and an attention-based multimodal Transformer [26], both of which integrate complementary information from different modalities within a unified feature space. For temporal modeling, LSTM [27] and Temporal Transformer [28] are selected as comparative methods to verify the effectiveness of explicit temporal dependency modeling in fraud behavior identification.

In terms of evaluation metrics, a comprehensive assessment is conducted from multiple perspectives, including classification performance, discriminative stability, and adaptability to class imbalance. Accuracy, precision, recall, matthews correlation coefficient (MCC), and F1 score are adopted as the primary classification metrics, which are computed as follows:

(8) $$\mathrm{Accuracy}=\frac{TP+TN}{TP+TN+FP+FN},$$

(9) $$\mathrm{Precision}=\frac{TP}{TP+FP},\;\mathrm{Recall}=\frac{TP}{TP+FN},$$

(10) $$F1=\frac{2\times \mathrm{Precision}\times \mathrm{Recall}}{\mathrm{Precision}+\mathrm{Recall}},$$

(11) $$\mathrm{MCC}=\frac{TP\times TN-FP\times FN}{\sqrt{\left(TP+FP)(TP+FN)(TN+FP)(TN+FN\right)}}.$$

Here, TP, TN, FP, and FN denote the numbers of true positives, true negatives, false positives, and false negatives, respectively. The joint use of these metrics enables a more comprehensive reflection of the overall performance, class discrimination capability, and stability of the model under imbalanced data conditions in financial fraud detection tasks.

### 4.3. Overall Comparison on Fraud Detection

This experiment is designed to systematically compare the overall discriminative capabilities of different types of models for short-video financial fraud detection under unified data splits and evaluation criteria, thereby verifying the necessity and effectiveness of multimodal and temporal modeling for this task. By incorporating representative methods based on textual modeling, visual modeling, multimodal fusion, and temporal modeling, the experiment covers diverse technical paradigms ranging from unimodal static discrimination to multimodal dynamic modeling, enabling a comprehensive assessment of model applicability in complex real-world scenarios.

**Figure 4:**
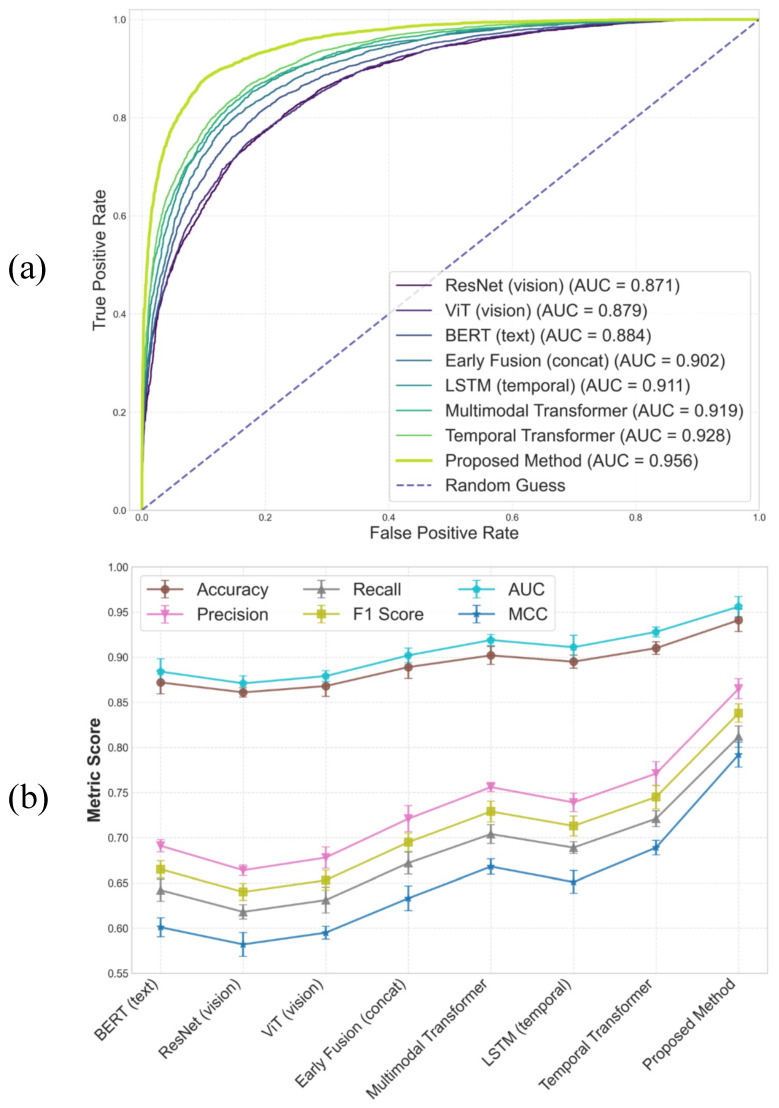
Overall performance visualization: (**a**) ROC curves and (**b**) key evaluation metrics across different models.

As shown in Table 2 and Figure 4, unimodal models exhibit relatively limited overall performance. BERT demonstrates advantages in textual semantic understanding; however, by relying solely on linguistic cues such as comments and marketing scripts, implicit visual and interaction information embedded in fraudulent behaviors cannot be effectively modeled, resulting in constrained recall and f1 scores. ResNet and ViT, as visual models, are capable of capturing visual cues related to scene structure, product presentation, and presenter behavior, yet their discriminative capacity remains limited in the absence of semantic and interaction context, making it difficult to distinguish videos with similar content but different underlying intentions. Compared with ResNet, ViT achieves slightly better performance, reflecting the potential advantage of self-attention architectures in modeling global relationships within complex visual scenes, although the overall improvement remains modest. With the introduction of multimodal fusion and temporal modeling capabilities, a clear upward trend in performance is observed. The early fusion method leverages complementary information from multiple modalities through simple feature concatenation, leading to consistent improvements over unimodal models across all metrics. However, since this fusion strategy is mathematically equivalent to a linear combination and lacks explicit modeling of inter-modal relationships and dynamic weighting, the performance gain remains limited. The multimodal transformer further enhances performance by modeling cross-modal interactions through attention mechanisms in a unified representation space, resulting in additional improvements in precision, recall, and auc. LSTM, as a classical temporal model, captures temporal dependencies through recurrent state transitions and outperforms multimodal models without explicit temporal structures, indicating that the temporal evolution of fraudulent behavior plays a critical role in overall discrimination. The temporal transformer further improves performance by directly modeling dependencies between arbitrary time steps via self-attention, thereby avoiding gradient attenuation issues associated with recurrent architectures. In contrast, the proposed method achieves significant performance gains across all metrics, with particularly notable advantages in recall, auc, and mcc, demonstrating not only stronger overall discriminative capability but also greater stability under imbalanced data conditions. Theoretically, this superiority stems from the alignment of multimodal signals under a unified timeline, combined with cross-modal attention and evolution modeling, which enables dynamic characterization of stage-specific fraud signals and cumulative risk trends, thereby yielding enhanced expressive power and generalization ability in complex short-video financial fraud scenarios.

### 4.4. Early-Stage Detection Performance

This experiment is designed to evaluate the capability of different models to identify financial fraud behaviors when only early-stage video content is available, thereby validating their effectiveness in risk assessment and early warning under incomplete information. By truncating the input sequence to the first 30% of video content, this experiment simulates early monitoring scenarios on real platforms, where content has not fully unfolded and interactions have not yet accumulated, allowing assessment of each model’s sensitivity to weak signals and initial anomalous patterns. Crucially, to strictly preclude any “future information” leakage (look-ahead bias) during early-stage inference, the temporal alignment process operates in a purely causal manner. The temporal mapping operators and alignment weights $\omega_{\tau ,i}^{m}$ at any given time step τ are computed strictly using the observed prefix sequence, ensuring no future context is accessed. Moreover, the rotary positional embeddings are anchored to absolute temporal indices, guaranteeing that the encoded representations of the first 30% of the sequence remain logically and mathematically consistent with those in the full-length sequence. This ensures our early-stage evaluation authentically reflects real-time, unidirectional risk prediction capabilities without implicitly exploiting late-stage patterns.

**Figure 5:**
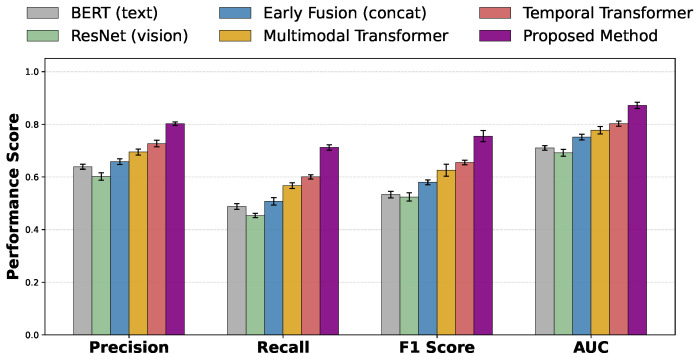
Performance comparison between the proposed method and baselines across core evaluation metrics.

As shown in Table 3 and Figure 5, the discriminative performance of unimodal models is significantly constrained in early-stage scenarios. Although BERT can capture certain persuasive scripts and semantic anomalies, its recall remains relatively low in the absence of subsequent comment diffusion and behavioral amplification, reflecting the instability of textual cues at early stages. ResNet relies exclusively on visual information and is sensitive to subtle anomalies in presenter actions or layout; however, such visual differences are often insufficiently pronounced at early stages, further limiting detection performance. Early fusion alleviates information scarcity to some extent by incorporating multiple modalities, yet its static fusion strategy restricts the ability to model varying discriminative contributions of different modalities during early stages. As explicit temporal modeling and cross-modal interaction capabilities are strengthened, performance in early detection tasks improves progressively. The multimodal transformer establishes associations among multimodal representations through attention mechanisms, enabling more effective integration of complementary cues from text and vision within limited time windows, resulting in clear improvements in precision, recall, and auc. The temporal transformer further introduces global temporal dependency modeling, allowing latent evolution trends to be inferred even from short sequences, highlighting the discriminative value of structural temporal information. In comparison, the proposed method achieves substantial performance advantages across all metrics, particularly in recall and f1, indicating enhanced sensitivity and stability in early-stage fraud perception. Theoretically, this advantage arises from multimodal alignment under a unified timeline and dynamic reinforcement of more discriminative modalities at early stages, enabling risk estimation to rely on latent evolution patterns rather than explicit late-stage bursts. This time-evolution–centric modeling paradigm allows reliable risk assessment even under incomplete information, aligning well with practical requirements for early intervention and proactive governance on short-video platforms.

### 4.5. Ablation Study of Key Components

This ablation experiment is conducted to systematically analyze the contribution of key components within the proposed model, thereby validating the rationality and necessity of the overall framework design. By selectively removing or modifying individual modules while keeping all other network structures and training configurations unchanged, the experiment clearly reveals the functional roles of different components in multimodal financial fraud detection.

**Figure 6:**
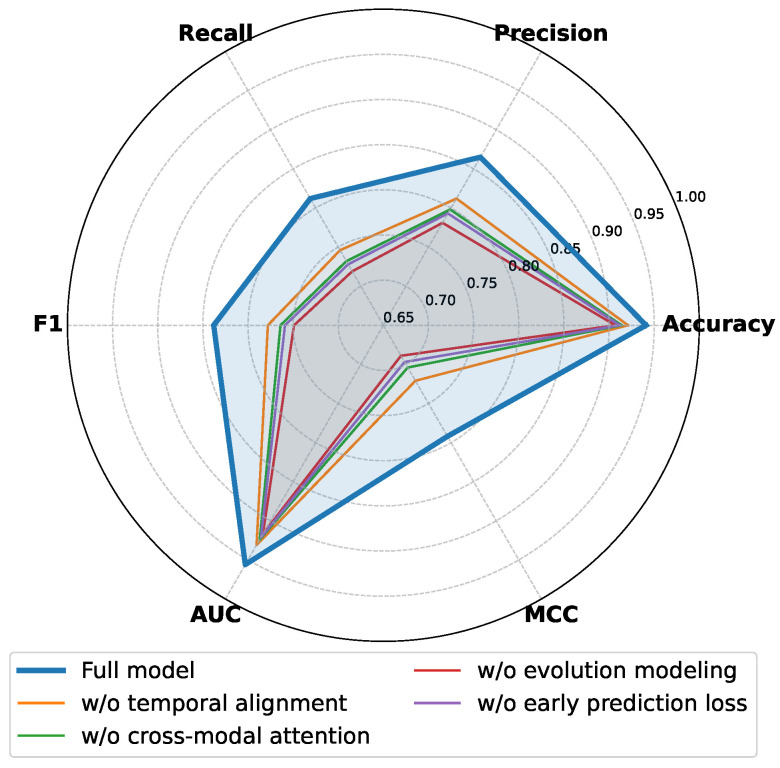
Radar chart comparing the full model and its ablated variants across multiple evaluation metrics.

As shown in Table 4 and Figure 6, the full model achieves the best performance across all evaluation metrics, indicating that complementary effects emerge when all components operate jointly. Removing the temporal alignment module leads to a noticeable performance degradation, particularly in recall, f1, and auc, which are closely related to risk coverage, suggesting that temporal misalignment weakens the joint expression of multimodal evidence for the same event. When cross-modal attention is removed, multimodal information can still be utilized, but the inability to dynamically adjust modality contributions across different temporal stages results in reduced discriminative capacity. The removal of evolution modeling further degrades overall performance, reflecting the limitation of relying solely on local or static temporal features to capture the cumulative progression of fraud behaviors. From a theoretical perspective, the observed performance variations can be explained by differences in representational capacity and structural constraints. Temporal alignment introduces a unified temporal reference in latent space, ensuring comparability of multimodal features at each time step and reducing representation noise caused by sampling discrepancies. Cross-modal attention performs dynamic weighting across modalities, effectively introducing time-varying adaptive combination coefficients that enhance expressiveness for non-stationary signals. Evolution modeling expands the temporal receptive field, enabling risk decisions to be based on holistic trajectories rather than isolated strong signals. The performance decline observed when early prediction loss is removed indicates that stage-wise supervision serves as an important regularizer during optimization, enforcing temporal consistency and monotonicity in risk estimation, thereby stabilizing training and improving generalization. Overall, the ablation results validate the complementarity of individual components and demonstrate that the complete framework is well suited to the multimodal and temporal characteristics of short-video financial fraud detection.

### 4.6. Ablation Study on Parameter Sharing Strategy

To empirically validate the effectiveness of the shared temporal encoding network and demonstrate the advantages of its inductive bias, an ablation experiment is conducted focusing on the parameter sharing strategy. Three distinct architectural variants are constructed and evaluated under identical training configurations. The first variant is the proposed shared encoder model, which enforces parameter sharing across all modalities throughout the entire network depth. The second variant utilizes separate encoders, where each modality is processed by an independent transformer encoder with identical depth and width, followed by the same late fusion mechanism. The third variant implements a partial sharing strategy, wherein the lower two transformer layers are shared across modalities to capture basic temporal alignments, while the top two layers remain modality-specific to preserve high-level distinct semantics.

The experimental results presented in Table 5 clearly indicate that the full parameter sharing strategy achieves the best performance across all evaluation metrics. The separate encoders variant yields the lowest performance among the three, suggesting that independent temporal modeling fails to constrain the heterogeneous signals into a functionally aligned semantic space, which consequently weakens the effectiveness of the subsequent cross-modal attention mechanism. The partial sharing variant provides a moderate improvement over the separate encoders, indicating that while sharing lower-level representations is beneficial, it is insufficient for resolving deep semantic misalignments. By enforcing identical temporal modeling operators across all layers, the proposed shared encoder introduces a strong inductive bias that successfully drives modality representations to converge into a common temporal subspace. This empirical evidence supports the theoretical claim that parameter sharing is a critical component for effectively capturing cross-modal temporal evolution patterns prior to dynamic fusion.

### 4.7. Ablation Study on Cross-Modal Fusion Mechanisms

To further validate the necessity of the proposed cross-modal temporal attention mechanism, an ablation experiment is conducted to compare it with a simpler alternative fusion strategy. In this experiment, the attention module is replaced by a multi-layer perceptron gating network. For the experimental settings, the gating network takes the concatenated multimodal features at each time step as input and maps them through two fully connected layers with an intermediate activation function to output a three-dimensional weight vector. A softmax function is then applied to generate normalized gating weights for the visual, audio, and textual modalities, which are subsequently used to compute the weighted sum of the modality representations. The rest of the network architecture, including the shared temporal encoding and the fraud evolution modeling modules, remains identical to ensure a fair comparison. Both models are trained under the exact same optimization configurations.

The experimental results presented in Table 6 demonstrate that the proposed cross-modal attention mechanism consistently outperforms the simpler MLP-based gating network across all evaluation metrics. While the MLP gating network achieves reasonable performance by learning a parameterized mapping from the concatenated features to modality weights, it lacks the dynamic interaction capability inherent to the attention mechanism. The attention module explicitly models the dot-product similarity between the global multimodal context query and the individual modality keys, allowing for more fine-grained and adaptive modality selection at each specific temporal stage. In complex financial fraud scenarios where the discriminative power of visual, audio, and textual cues fluctuates rapidly over time, the static parameterized mapping of the MLP struggles to fully capture these non-stationary temporal dynamics, resulting in a noticeable drop in recall and overall F1 score. Therefore, the attention-based fusion is proven to be a more effective structural choice for this task.

### 4.8. Robustness Analysis Under Modality Missing and Degradation

To comprehensively evaluate the robustness of the proposed framework against real-world deployment constraints, a series of modality dropout stress tests and degradation experiments are conducted. In practical scenarios, short-video platforms frequently encounter situations such as disabled comment sections, muted video playbacks, or highly noisy audio streams. To simulate these conditions, we introduce a modality dropout strategy during the training phase, where each modality is randomly masked with a certain probability, forcing the shared temporal encoder and cross-modal attention module to learn adaptive compensatory representations. During inference, we evaluate the model under several stringent conditions: complete absence of the textual modality to simulate missing comments, complete absence of the audio modality to simulate muted playbacks, partial absence of comments where fifty percent of the textual sequence is randomly dropped, and audio degradation where signal-to-noise ratio is significantly lowered by adding Gaussian noise to the speech stream. All other hyperparameters and temporal alignment settings remain strictly identical to the main experiments to ensure a fair comparison.

The experimental results presented in Table 7 demonstrate that the proposed framework maintains a highly stable discriminative capability even under severe data degradation and missing modality conditions. While the complete removal of textual or audio information inevitably leads to a performance decline compared to the full-modality upper bound, the model does not suffer from catastrophic failure. Specifically, under the partial comments and audio degradation settings, the F1 score only experiences a marginal drop, indicating strong resilience to noisy and incomplete sequential inputs. This robustness is fundamentally attributed to the cross-modal temporal attention fusion module. Rather than relying on rigid feature concatenation which can become brittle when an input branch is zeroed out, the attention mechanism dynamically redistributes its weighting distribution based on the available active modalities. When a specific modality is missing or highly corrupted, the module autonomously suppresses its attention weight and amplifies the reliance on the remaining intact modalities at each temporal stage. This empirical evidence firmly validates the adaptive nature of our fusion architecture and confirms its practical viability for deployment in unpredictable real-world platform environments.

### 4.9. Extension to Temporal Graph Modality

To systematically address the limitations regarding cross-account and cross-platform coordinated manipulation, we introduce an additional experimental setting that integrates temporal graph signals as a fourth modality. In this setup, a dynamic heterogeneous graph is constructed for each video, encompassing user-video interactions and comment-reply structural relations over time. At each time step, a temporal graph attention network is employed to aggregate local neighborhood structures, yielding a sequence of graph-based temporal embeddings. These structural embeddings are then seamlessly integrated into our cross-modal temporal attention fusion module alongside the visual, audio, and textual representations. To validate the effectiveness of this extension, we compare the extended four-modality framework against a strong purely structural baseline, namely the Temporal Graph Attention Network applied exclusively to the interaction graph, as well as our original three-modality proposed method. All models are trained and evaluated under the identical chronological data splitting strategy and optimization configurations as detailed in previous sections.

The experimental results presented in Table 8 clearly demonstrate that integrating temporal graph signals yields a substantial performance improvement across all evaluation metrics. The pure Temporal GNN baseline achieves reasonable discrimination based solely on interaction topologies, yet it falls short of the multimodal approaches due to its inability to perceive the semantic and visual persuasive context embedded in the video content. Meanwhile, the extended four-modality framework outperforms the original three-modality model, achieving notable gains particularly in recall and the overall F1 score. This improvement indicates that structural graph signals act as a powerful complementary feature, effectively capturing latent coordinated group behaviors such as synchronized fake reviews and anomalous interaction bursts that are otherwise invisible when observing content sequences in isolation. These findings firmly validate that the proposed temporal perception architecture possesses high extensibility and that the incorporation of relational topologies significantly strengthens the robustness of financial fraud detection in online platforms.

### 4.10. Cross-Platform Portability and Generalization Analysis

To empirically validate the portability and generalization capabilities of the proposed multimodal temporal perception framework across different online environments, a comprehensive leave-one-platform-out cross-validation experiment is conducted. Given that our dataset is compiled from several mainstream public short-video and content community platforms, specifically Douyin, Kuaishou, and Xiaohongshu, we systematically isolate each platform during the training phase. In this setup, the model is trained exclusively on data from two platforms and then evaluated entirely on the unseen third platform. This rigorous evaluation strategy simulates a zero-shot deployment scenario on a novel platform, thereby strictly assessing whether the model learns universal temporal fraud evolution patterns or merely overfits to platform-specific visual artifacts and interface biases. All hyperparameter settings, temporal alignment mechanisms, and optimization strategies remain completely identical to the main experiments to ensure a highly controlled and fair comparison.

The experimental results presented in Table 9 strongly demonstrate the robust cross-platform generalization capability of the proposed method. When evaluated on entirely unseen platforms, the framework consistently maintains a high level of discriminative performance, with the AUC remaining above 0.93 and the accuracy exceeding 0.91 across all test cases. While there is a slight, expected degradation in performance metrics compared to the fully mixed training scenario due to inherent cross-platform distribution shifts, the overall effectiveness does not experience catastrophic failure. The model performs slightly better when transferred to Douyin and Kuaishou compared to Xiaohongshu, which can be attributed to the higher similarity in their short-video interaction mechanisms and user behavioral dynamics. Ultimately, these results firmly substantiate the declarative claims of portability, proving that the cross-modal temporal attention and evolution modeling components successfully capture the fundamental, platform-agnostic evolutionary patterns of financial fraud.

### 4.11. Sensitivity and Calibration Analysis of Evolution Modeling

To comprehensively address the sensitivity and reliability of the fraud evolution modeling module, we design three interconnected evaluation settings. First, we conduct a sensitivity analysis on the gate activation threshold θ and the straight-through estimator (STE) temperature parameter, systematically varying θ from 0.3 to 0.7 and the temperature from 0.1 to 2.0 to observe their impact on overall detection performance. Second, we investigate whether the monotonicity constraint inadvertently inflates the False Positive Rate (FPR) on benign or highly popular content over time. We evaluate the FPR at the 30%, 50%, and 100% temporal truncation points for models trained with and without the temporal monotonicity loss. Third, we extract the predicted risk probabilities at these specific time percentiles and compute reliability curves (calibration plots) to verify whether the model outputs represent well-calibrated risk trajectories rather than unscaled confidence logits. All evaluations use the identical temporal split testing set as previous experiments.

**Figure 7:**
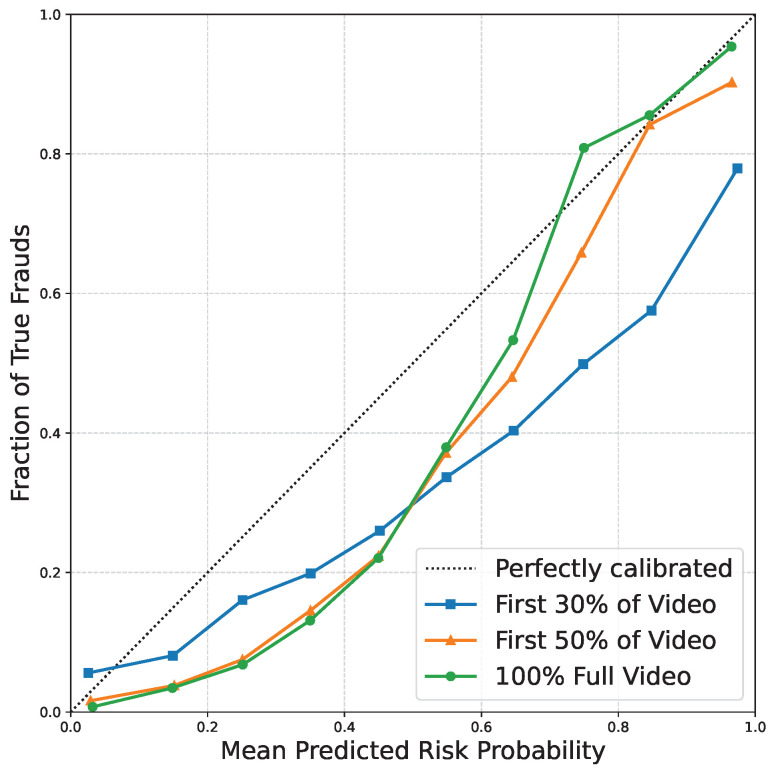
Calibration plots at different temporal stages.

The experimental results presented in Table 10 and Table 11 and Figure 7 validate the robustness and safety of the evolution modeling module. The sensitivity analysis demonstrates that the model maintains highly stable F1 scores across a moderate range of hyperparameters, achieving optimal routing behavior when the threshold is 0.5 and the temperature is around 0.5. Extreme temperatures cause the gating distribution to become either too rigid or entirely uniform, marginally degrading the expert selection efficacy. Furthermore, the FPR analysis definitively shows that the temporal monotonicity constraint does not cause a detrimental accumulation of false positives on benign content. Because benign videos typically exhibit consistently low risk signals from the outset, constraining the predicted risk sequence to be non-decreasing primarily prevents unnatural algorithmic score fluctuations, rather than artificially forcing a false escalation of risk over time. Finally, the generated calibration plots for different temporal stages reveal that the predicted risk probabilities closely align with the perfectly calibrated diagonal line. This excellent calibration confirms that the stage-wise risk scores outputted by the model can be reliably interpreted as true confidence metrics for early warning interventions.

### 4.12. Analysis of Cross-Modal Attention Stability and Softmax Temperature

To thoroughly evaluate the sensitivity of our cross-modal attention weights to input feature scaling and to ensure gradient stability over long time sequences, we designed an ablation study focusing on attention score normalization and variance collapse. Specifically, we introduce a temperature scaling parameter τ into the softmax normalization step of our cross-modal temporal attention mechanism, modifying the original formulation to scale the dot products before applying the softmax function. In our experimental setup, we vary the temperature parameter $\tau \in \{0.5,1.0,\sqrt{d_{k}},2.0\}$, where $d_{k}$ is the dimensionality of the key vectors, and τ=1.0 represents the unscaled baseline. To assess gradient stability and variance collapse, we monitor the average variance of the pre-softmax logits and the $L_{2}$ norm of the gradients propagated through the attention layer during training on long sequences exceeding 1000 frames. Furthermore, we evaluate the overall downstream performance metrics, including F1 score and AUC, to determine the optimal scaling strategy that preserves the necessary phase shifts modeled by the positional encodings across different modalities.

The experimental results demonstrate a clear vulnerability to variance collapse when the attention scores are left unscaled. As shown in Table 12, utilizing a standard unscaled softmax (τ=1.0) or a reduced temperature (τ=0.5) results in a massive inflation of logit variance, which sequentially pushes the softmax function into regions with extremely small gradients, evidenced by the critically low $L_{2}$ gradient norms. This vanishing gradient phenomenon severely destabilizes the training process for long temporal sequences, preventing the model from effectively learning long-range cross-modal dependencies. Conversely, applying the attention score normalization with $\tau =\sqrt{d_{k}}$ successfully regularizes the logit variance close to unit variance, ensuring robust gradient flow and preventing the attention distribution from collapsing into one-hot representations. This normalization also profoundly impacts the positional encodings; by restricting the magnitude of the attention logits, the relative phase shifts embedded by the positional encodings are preserved without being washed out by extreme feature scalings. Consequently, the scaled dot-product attention yields the most stable convergence and achieves the highest F1 and AUC scores, confirming its necessity for modeling prolonged multimodal sequences.

### 4.13. Discussion

In real-world short-video and live-str eaming e-commerce scenarios, financial fraud rarely manifests as a single abrupt event, but rather evolves through gradual content priming, persuasive scripting, and progressively intensified user interactions. The proposed multimodal temporal perception framework exhibits strong practical relevance under such conditions. For instance, in live-streamed financial courses or investment recommendation videos, presenters often establish trust during early stages through neutral market analysis or experience sharing, gradually introduce high-return implications or ambiguous promises in intermediate stages, and finally create urgency through limited-time offers, herd-effect comments, or incentivized interactions. Conventional methods based on single-modality cues or isolated frames struggle to distinguish such content from legitimate marketing at early stages, whereas the proposed approach can capture latent risk signals during initial publication or broadcast by jointly perceiving variations in speech prosody, comment dynamics, and visual content, thereby providing more proactive early warnings for platforms. From the perspective of platform governance, the proposed method can be deployed as a front-end perception module within content moderation and risk control systems. When a sustained increase in predicted risk is detected at early stages, stricter manual review, reduced recommendation exposure, or interaction restrictions can be triggered, suppressing fraudulent content dissemination without adversely affecting legitimate creators. In terms of user experience, such temporally aware risk identification mechanisms help avoid reactive, post-hoc enforcement, reducing the likelihood of users being misled or financially harmed without prior awareness. Moreover, joint modeling of multimodal evidence enhances interpretability, enabling platforms to attribute detected risks to factors such as abnormal scripting, sudden interaction surges, or inconsistencies between visual presentation and content, thereby supporting informed follow-up actions. In cross-platform application scenarios, differences in interaction mechanisms, content styles, and user behavior patterns exist among short-video and content-sharing platforms, yet the core evolution logic of financial fraud strategies remains largely consistent. By emphasizing multimodal perception under a unified timeline, the proposed method focuses on behavioral patterns rather than platform-specific superficial features, facilitating adaptation to new platforms or content formats. For example, when fraudsters alter linguistic expressions or visual packaging, anomalies in temporal rhythm and interaction patterns may still be captured, reducing vulnerability to strategy drift. Overall, the proposed approach not only improves detection accuracy in practical business settings, but also introduces a process-oriented, early-intervention–focused technical pathway for platform governance, contributing to the maintenance of a healthier digital content ecosystem.

### 4.14. Limitation and Future Work

Although the proposed multimodal temporal financial fraud perception method achieves promising experimental performance in short-video and live-streaming e-commerce scenarios, several aspects merit further investigation and improvement. First, the model exhibits a degree of dependence on data completeness during multimodal joint modeling; when certain modalities are persistently missing or severely degraded, temporal alignment and cross-modal fusion effectiveness may be affected, which remains a practical challenge in real platform environments. Second, although temporal modeling and early risk prediction enhance sensitivity to progressive fraud behaviors, extremely short-lived or abrupt fraud content may still require finer-grained behavioral features or external signals for stable identification. Moreover, the current approach primarily characterizes fraud evolution from content and interaction perspectives, leaving room for extending structural modeling capabilities to address more complex cross-account and cross-platform coordinated manipulation behaviors. Future work may explore more flexible modality-adaptive mechanisms, enabling dynamic adjustment of modeling strategies under varying modality availability conditions to enhance robustness in real-world deployments. Incorporating long-term behavioral trajectories across videos and accounts into a unified modeling framework could further improve the characterization of organized fraud and long-term manipulation patterns. Additionally, integrating causal analysis or explainable learning techniques to provide deeper mechanistic interpretations of risk predictions may enhance acceptability in regulatory and compliance contexts. Finally, subject to privacy and compliance constraints, validation and transfer on larger-scale, cross-regional datasets would further promote the practical adoption of multimodal temporal fraud perception methods on real-world platforms.

## 5. Conclusions

With the rapid rise of short-video platforms and live-streaming e-commerce, financial fraud has become increasingly covert, characterized by strong temporal dependencies and complex multimodal coupling. To address this, we propose a multimodal temporal perception framework that jointly models visual, audio, and textual streams along a unified timeline to characterize the dynamic evolution of fraudulent behaviors. By integrating temporal alignment, cross-modal attention fusion, and stage-aware risk prediction, our approach effectively captures non-stationary fraud patterns from weak early signals to concentrated outbreaks. Extensive experiments demonstrate that the proposed method consistently outperforms existing text, vision, and temporal baselines, achieving over 0.94 accuracy and maintaining robust early-warning capabilities even when utilizing only the first 30% of video content. Despite these strong results, the framework’s dense cross-modal attention introduces substantial computational overhead, which may pose latency challenges for real-time deployment. Furthermore, its performance relies on multimodal signal completeness and remains constrained when facing entirely novel, zero-day fraud patterns. Ultimately, this work establishes a highly effective paradigm for early and accurate financial fraud detection in dynamic media scenarios, while highlighting essential future research directions towar.

## Figures and Tables

**Table 1:** Statistics of the multimodal short-video financial fraud dataset.

Data Modality	Data Scale	Temporal Granularity	Main Characteristics
Short-video content	18,742	Video-level/frame-level	Visual scenes, transitions, product presentation
Audio speech streams	18,742	Continuous time series	Intonation, rhythm, emotional variation
Textual information	2,316,584	Comment time series	Semantic content, sentiment, persuasive patterns
Fraud samples	3964	Video-level	Fraudulent marketing behaviors
Non-fraud samples	14,778	Video-level	Normal marketing behaviors

**Table 2:** Overall performance comparison on short-video financial fraud detection (Mean ± Std across 5 seeds).

Method	Accuracy	Precision	Recall	F1	AUC	MCC	Params (M)	Latency (ms)
BERT (text)	0.872±0.006	0.691±0.008	0.642±0.007	0.665±0.008	0.884±0.005	0.601±0.007	110.5	28.4±1.2
ResNet (vision)	0.861±0.007	0.664±0.009	0.618±0.008	0.640±0.007	0.871±0.006	0.582±0.008	25.6	18.2±0.8
ViT (vision)	0.868±0.005	0.678±0.007	0.631±0.006	0.653±0.005	0.879±0.004	0.595±0.006	86.4	24.6±1.0
Early Fusion (concat)	0.889±0.005	0.721±0.006	0.672±0.007	0.695±0.006	0.902±0.005	0.633±0.005	225.3	52.8±1.5
Multimodal Transformer	0.902±0.004	0.756±0.005	0.704±0.006	0.729±0.005	0.919±0.004	0.668±0.006	248.7	68.5±1.8
LSTM (temporal)	0.895±0.006	0.739±0.007	0.689±0.008	0.713±0.006	0.911±0.005	0.651±0.007	230.1	58.3±1.4
Temporal Transformer	0.910±0.004	0.771±0.005	0.721±0.004	0.745±0.004	0.928±0.003	0.689±0.005	245.2	72.1±2.1
Proposed Method	0.941±0.003	0.865±0.004	0.812±0.005	0.838±0.004	0.956±0.002	0.792±0.004	185.4	62.7±1.6

**Table 3:** Early-stage fraud detection performance under temporal truncation (first 30% of video, Mean ± Std across 5 seeds).

Method	Precision	Recall	F1	AUC
BERT (text)	0.632±0.009	0.481±0.008	0.546±0.008	0.721±0.007
ResNet (vision)	0.604±0.011	0.458±0.009	0.521±0.010	0.704±0.008
Early Fusion (concat)	0.661±0.008	0.512±0.007	0.577±0.007	0.748±0.006
Multimodal Transformer	0.701±0.006	0.561±0.008	0.623±0.006	0.781±0.005
Temporal Transformer	0.726±0.005	0.598±0.006	0.656±0.005	0.806±0.004
Proposed Method	0.812±0.004	0.704±0.005	0.754±0.004	0.869±0.003

**Table 4:** Ablation study of key components in the proposed framework.

Variant	Accuracy	Precision	Recall	F1	AUC	MCC
Full model	0.941	0.865	0.812	0.838	0.956	0.792
w/o temporal alignment	0.921	0.812	0.746	0.778	0.931	0.721
w/o cross-modal attention	0.914	0.798	0.732	0.764	0.924	0.704
w/o evolution modeling	0.907	0.781	0.719	0.749	0.917	0.689
w/o early prediction loss	0.912	0.793	0.728	0.759	0.921	0.697

**Table 5:** Performance comparison of different parameter sharing strategies.

Method	Accuracy	Precision	Recall	F1	AUC	MCC
Separate encoders	0.918	0.805	0.741	0.772	0.932	0.715
Partial sharing	0.925	0.822	0.765	0.792	0.941	0.738
Shared encoder (Proposed)	0.941	0.865	0.812	0.838	0.956	0.792

**Table 6:** Performance comparison between different cross-modal fusion mechanisms.

Fusion Mechanism	Accuracy	Precision	Recall	F1	AUC	MCC
MLP Gating Network	0.923	0.816	0.755	0.784	0.935	0.731
Cross-Modal Attention (Proposed)	0.941	0.865	0.812	0.838	0.956	0.792

**Table 7:** Robustness evaluation under modality missing and degradation conditions.

Condition	Accuracy	Precision	Recall	F1	AUC	MCC
Full Modalities (Upper Bound)	0.941	0.865	0.812	0.838	0.956	0.792
Partial Comments (50% missing)	0.933	0.842	0.791	0.816	0.945	0.763
Audio Degradation (Noisy ASR)	0.928	0.831	0.784	0.807	0.938	0.751
Missing Textual Modality	0.905	0.785	0.732	0.758	0.914	0.692
Missing Audio Modality	0.912	0.794	0.745	0.769	0.921	0.708

**Table 8:** Performance comparison incorporating temporal graph signals.

Method	Accuracy	Precision	Recall	F1	AUC	MCC
Temporal GNN Baseline	0.892	0.735	0.681	0.707	0.905	0.642
Proposed Method (3 Modalities)	0.941	0.865	0.812	0.838	0.956	0.792
Extended Framework (4 Modalities)	0.953	0.884	0.845	0.864	0.968	0.825

**Table 9:** Leave-one-platform-out cross-validation performance for portability analysis.

Target Platform (Testing)	Accuracy	Precision	Recall	F1	AUC	MCC
Test on Douyin	0.931	0.842	0.795	0.817	0.948	0.765
Test on Kuaishou	0.925	0.835	0.781	0.807	0.941	0.752
Test on Xiaohongshu	0.918	0.816	0.764	0.789	0.932	0.738

**Table 10:** Impact of temporal monotonicity constraint on False Positive Rate (FPR) for benign content.

Method	FPR at 30%	FPR at 50%	FPR at 100%
Without Monotonicity Loss	0.042	0.048	0.051
With Monotonicity Loss (Proposed)	0.045	0.047	0.054

**Table 11:** Sensitivity analysis of the F1 score with respect to threshold θ and STE temperature.

Threshold (θ)	Temp = 0.1	Temp = 0.5	Temp = 1.0	Temp = 2.0
θ=0.3	0.812	0.824	0.819	0.801
θ=0.5 (Proposed)	0.827	0.838	0.832	0.815
θ=0.7	0.805	0.818	0.811	0.793

**Table 12:** Impact of softmax temperature scaling (τ) on attention logit variance, gradient stability, and overall performance.

Temperature (τ)	Logit Variance	Gradient Norm ($L_{2}$)	F1	AUC
0.5	142.35	0.02	0.712	0.825
1.0 (Unscaled)	85.62	0.15	0.785	0.892
$\sqrt{d_{k}}$ (Scaled)	1.05	2.84	0.841	0.958
2.0	0.42	3.12	0.810	0.915

## Data Availability

The data presented in this study are available on request from the corresponding author.
